# Supplementary material for: Bovine Serum Albumin as a Platform for Designing Biologically Active Nanocarriers—Experimental and Computational Studies
Source: Int J Mol Sci. 2023 Dec 19;25(1):37. doi: 10.3390/ijms25010037 (PMC10778598; doi:10.3390/ijms25010037)
Supplement: Supplementary file 1 [file ijms-25-00037-s001.zip › ijms-2765035-supplementary.pdf]

Supplementary Materials

BSA as a platform for designing biologically active nanocarriers– experimental and computational studies

Olga Adamczyk[1], Magdalena Szota[2], Kamil Rakowski[2], Magdalena Prochownik[1], Daniel Doveiko[3], Yu Chen[3], Barbara Jachimska[2*]

[1]Department of Materials Engineering, Faculty of Materials Engineering and Physics, Cracow University of Technology, Krakow, Poland

[2]Jerzy Haber Institute of Catalysis and Surface Chemistry Polish Academy of Sciences, Krakow, Poland

[3]Department of Physics, University of Strathclyde, Glasgow G4 0NG, United Kingdom

*Correspondence: barbara.jachimska@ikifp.edu.pl

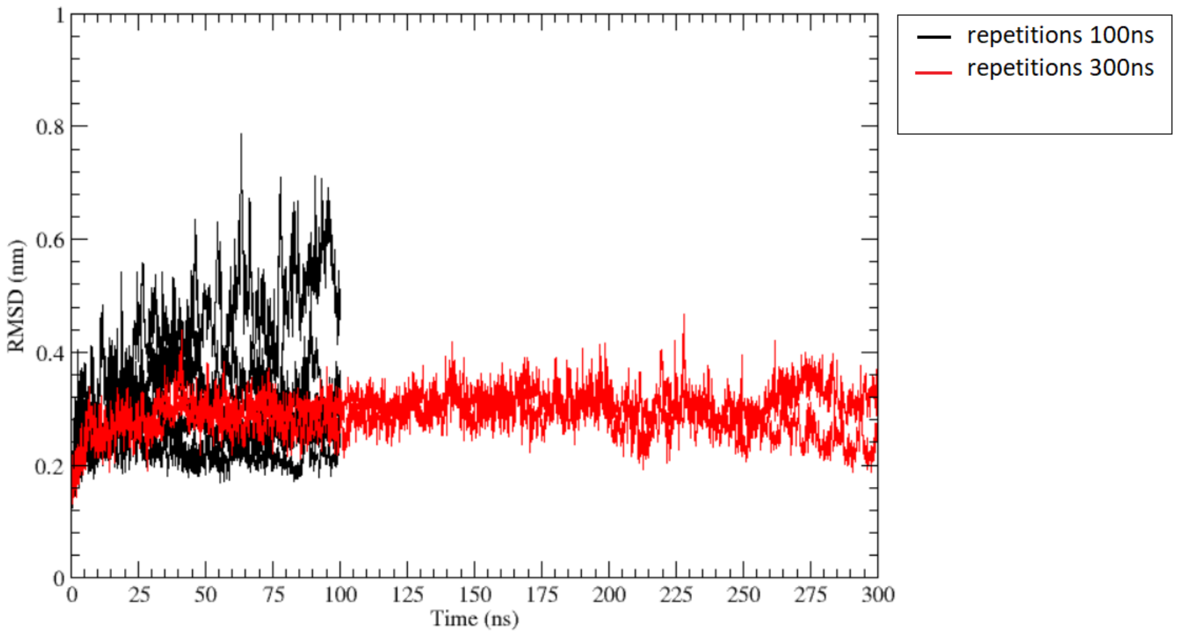

Figure. S1 Comparison of conformational complexity of all repeats, RMSD based on Cα-BSA carbon movements, 100ns (black curves), 300ns (red curves)

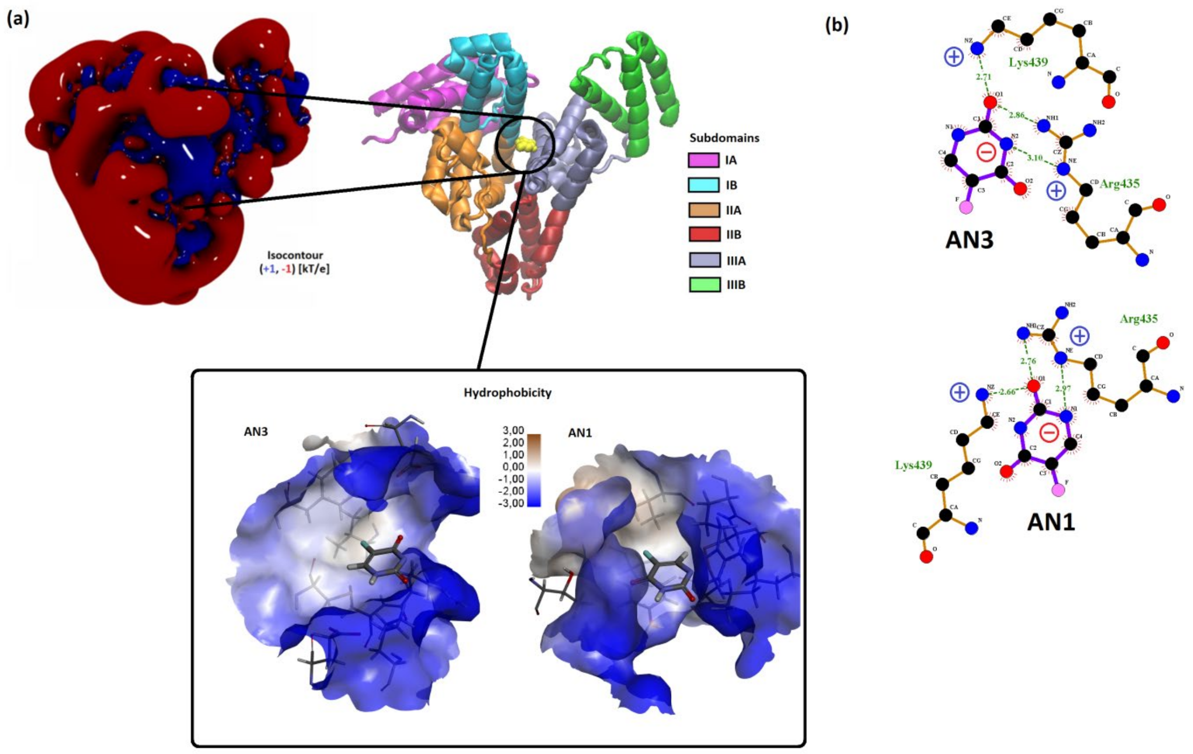

Figure S2. Comparison of affinity of tautomers to BSA subdomains after simulations, (a) visualization of the most stable location of AN1 and AN3 (Arg435, Lys439, Tyr451), (b) interaction diagrams of the most representative AN1* and AN3* complexes.

&general

sys_name="BSA_DRUG",

startframe=1508,

endframe=2942,

interval=10,

temperature=298.15,

PBRadii=6,

forcefields="oldff/leaprc.ff99SB,leaprc.gaff2"

/

&pb

indi=1, **exdi=**80, **istrng=**0.010, **radiopt=**0, **npbopt=**0,**inp=**2,

/

Figure S3. The gmx_MMPBSA script that determines the end state energy. Example of the most stable complex BSA-AN3

(a)

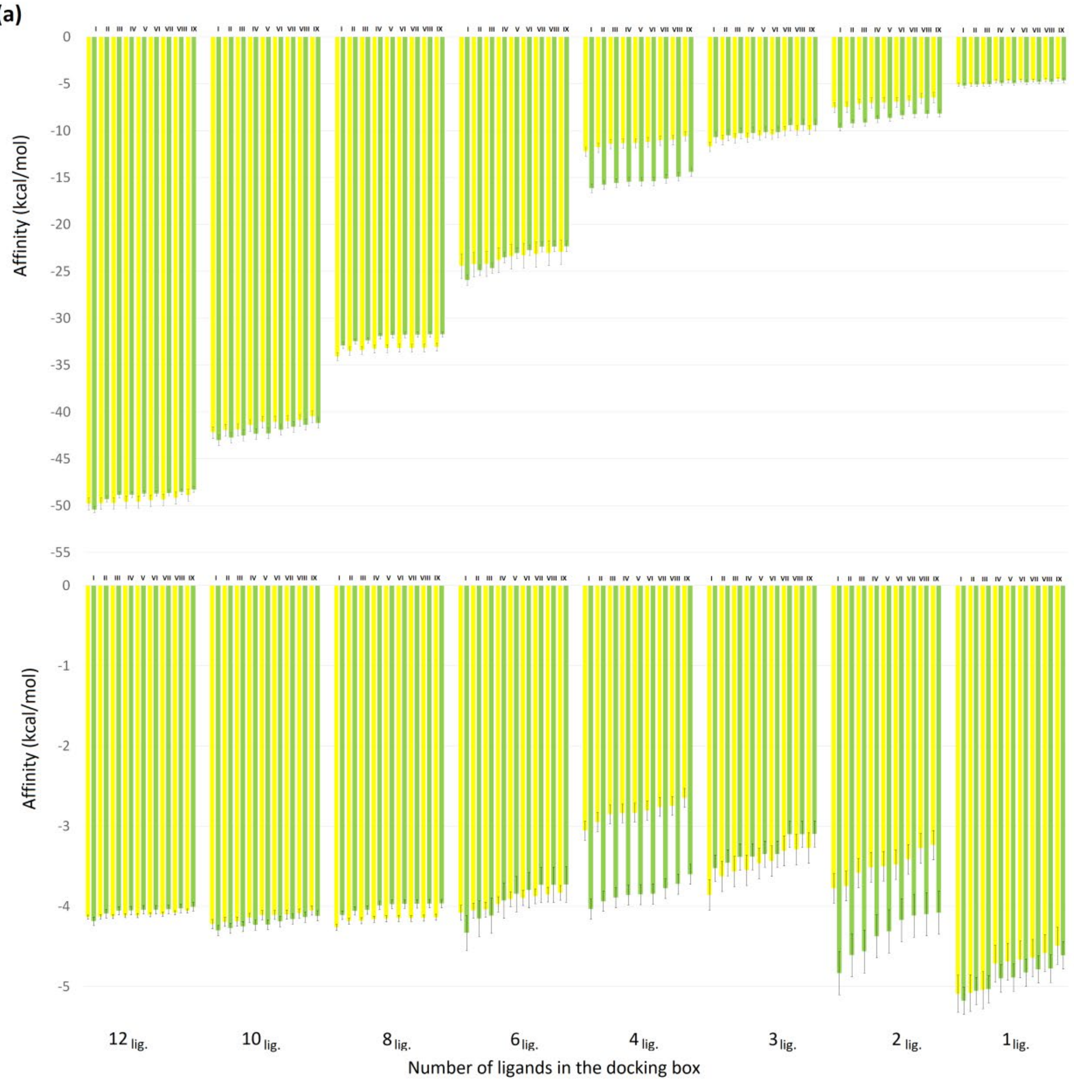

(b)

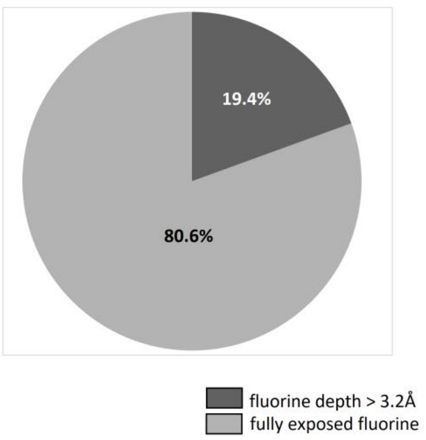

Figure S4. Summary of AutoDock Vina docking results, (a) comparison of Gibbs free energy changes for BSA/tautomer complexes as a function of the number of docked tautomers, (top graph), comparison of Gibbs free energy changes for complexes after conversion of energy per molecule (bottom graph; (b) total average depth distribution of fluorine atoms for docking complexes with twelve tautomer molecules.

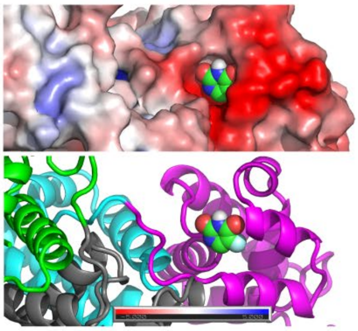

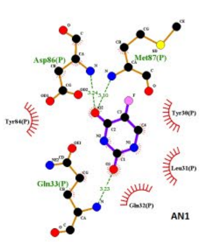

-5.079kcal/mol

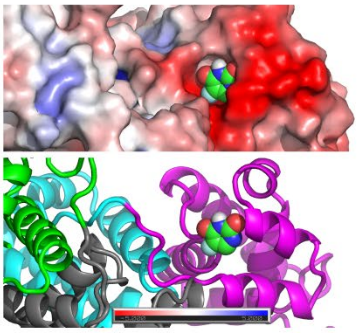

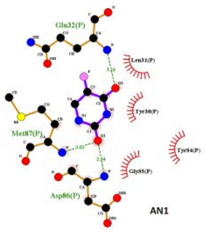

-5.043kcal/mol

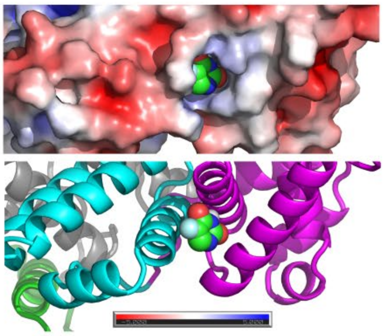

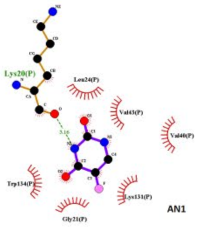

-4.713kcal/mol

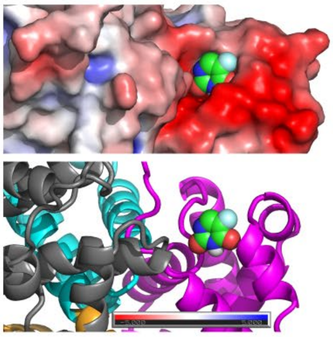

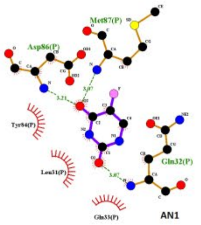

-4.689kcal/mol

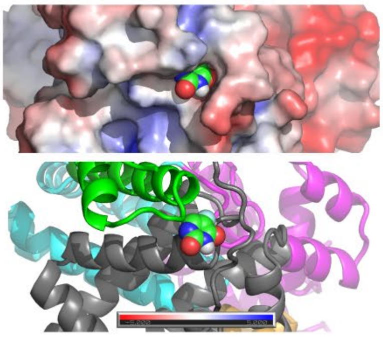

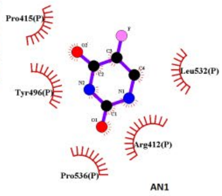

-4.662kcal/mol

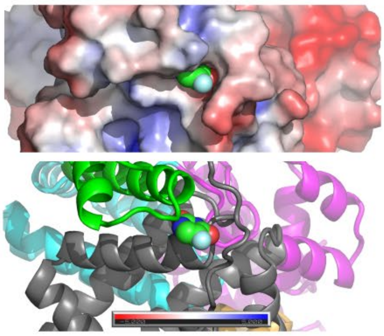

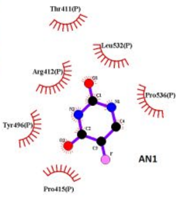

-4.641kcal/mol

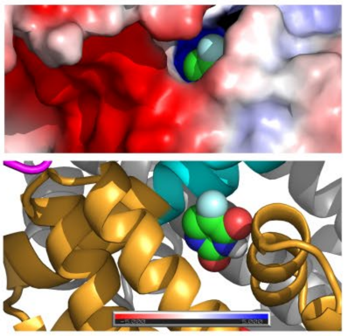

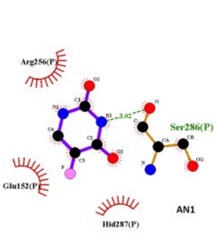

-4.582kcal/mol

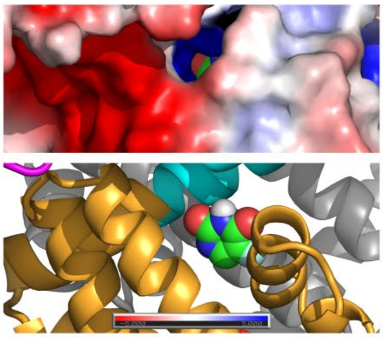

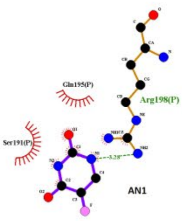

-4.491kcal/mol

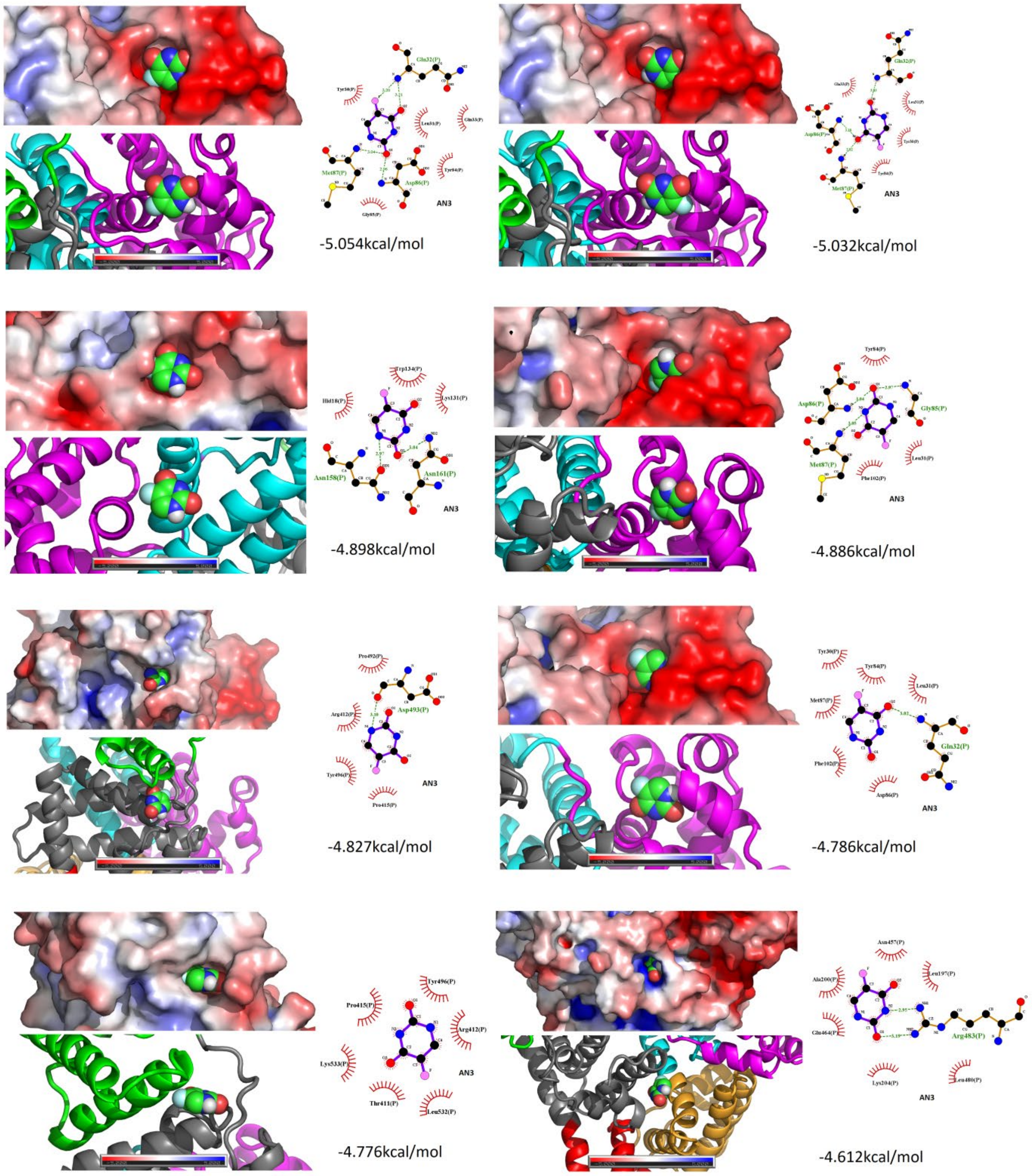

Figure S5. The result of docking a single molecule across the BSA volume, the other eight most likely configurations, BSA-AN1 and BSA-AN3, respectively. The electrostatic potential distribution on the VdW surface, the position in the subdomain on the backbone model, the interaction diagram, and the Gibbs free energy of the BSA-tautomer complex are marked, with a standard deviation within the group for AN1 and AN3 of ±0.230 kcal/mol and ± 0.171kcal/mol, respectively.
